# Role of Hydrogen Bonding in Green Fluorescent Protein-like Chromophore Emission

**DOI:** 10.1038/s41598-019-47660-0

**Published:** 2019-08-12

**Authors:** Li Yang, Shifeng Nian, Guozhen Zhang, Edward Sharman, Hui Miao, Xuepeng Zhang, Xiaofeng Chen, Yi Luo, Jun Jiang

**Affiliations:** 1 Institutes of Physical Science and Information Technology, Anhui University, Hefei, Anhui 230601 P. R. China; 2 Hefei National Laboratory for Physical Sciences at the Microscale, iChEM (Collaborative Innovation Centre of Chemistry for Energy Materials), CAS Centre for Excellence in Nanoscience, Department of Chemistry and Materials Science, University of Science and Technology of China, Hefei, Anhui 230026 China; 3 Department of Environmental Science and Engineering, College of Life and Environmental Science, Shanghai Normal University, Shanghai, 200234 China; 4 Department of Neurology, University of California, Irvine, California, 92697 United States

**Keywords:** Computational chemistry, Single-molecule fluorescence

## Abstract

The fluorescence emission from green fluorescent protein (GFP) is known to be heavily influenced by hydrogen bonding between the core fluorophore and the surrounding side chains or water molecules. Yet how to utilize this feature for modulating the fluorescence of GFP chromophore or GFP-like fluorophore still remains elusive. Here we present theoretical calculations to predict how hydrogen bonding could influence the excited states of the GFP-like fluorophores. These studies provide both a new perspective for understanding the photophysical properties of GFP as well as a solid basis for the rational design of GFP-based fluorophores.

## Introduction

The green fluorescent protein (GFP) has been an indispensable tool for bioimaging and bioengineering since it was discovered in the jellyfish *Aequorea victoria*^1–4^. Tagged with GFP, living cells and tissues can be monitored via fluorescence microscopy non-invasively^5^. GFP is a 238-aa protein with a molecular weight of about 26.9 kDa. The core of GFP, 4-(4-hydroxybenzylidene)-1,2-dimethyl-imidazolinone (p-HOBDI), is enclosed at the centre of an 11-stranded β-barrel, and spontaneously forms a complex by binding to amino acid residues Ser65-Tyr66-Gly67 after a multistep reaction^6^. The remaining residues fold into a host scaffold or matrix, providing an intricate microenvironment which is essential for keeping the fluorophore HOBDI highly emissive^7^.

Given that the synthetic replication of the three-residue-led moiety with exactly the same chemical structure as *p*-HOBDI only produces weak to no fluorescence in both solutions and polymer films, it is apparent that the key to the highly emissive state relies on the specificity of the surrounding protein matrix^8^. A generally accepted notion is that the protein skeleton increases the quantum yield of the fluorophore by restraining its rotational freedom^9,10^. In addition, conformations in the ground/excited state^11^, intermolecular interactions such as electrostatic interactions, hydrogen bond formation, π-π stacking, Van der Waals forces, medium viscosity, as well as many other parameters have been shown to influence the rate of non-radiative decay^12–16^. The degenerate contributions make it difficult to cast deep insights to tune the luminescence properties. As a result, it is truly essential to modulate the fluorescence of the GFP and GFP-like chromophore at the molecular and atomic level through theoretical understanding.

Among better-studied aspects of GFP, the equilibrium between the neutral and ionized states of the fluorophore is regulated by a hydrogen bonding network^17^. It is well documented that the hydrogen bonding interactions between the chromophore and neighbouring side chains or water molecules could significantly affect the photochemical process. The excited-state proton transfer, which is the dynamics of the hydrogen bonding would contribute to generate an intense main fluorescence emission band^18^. Grigorenko *et al*.^19^ simulated the proton transfer from the neutral or ionized structure to the surrounding amino acid side chains, demonstrating the significant role of hydrogen bonding network on the stability and emission of the chromophore. Oltrogge and Boxer *et al*.^20^ have verified the significance of low-barrier hydrogen bonding in color tuning of chromophore fluorescence by modifying the acidity of halide-substituted chromophores.

Inspired by this, in our work, hydrogen bonding is the key to precisely tune the emission of the GFP-like chromophore. Wherein, hydrogen and proton are chosen as the bridge to form hydrogen bonding. It has been shown that hydrogen bonding network could induce the proton travels via water and Ser205 to Glu222 during photoexcitation^21^. Furthermore, Fujisawa *et al*.^22^ have confirmed that the hydrogen-bond chain chromophore-water-Ser205-Glu222 is dedicated to the whole intermolecular and inter-residue vibrational modes in the excited-state structural evolution. And considering that the practical chromophore (for instance, the native GFP chromophore constrained in a protein cage) normally does not emit fluorescence in absence of water, we also investigated the role of water in forming an essential hydrogen-bond network with hydrogen and protons.

**Figure 1:**
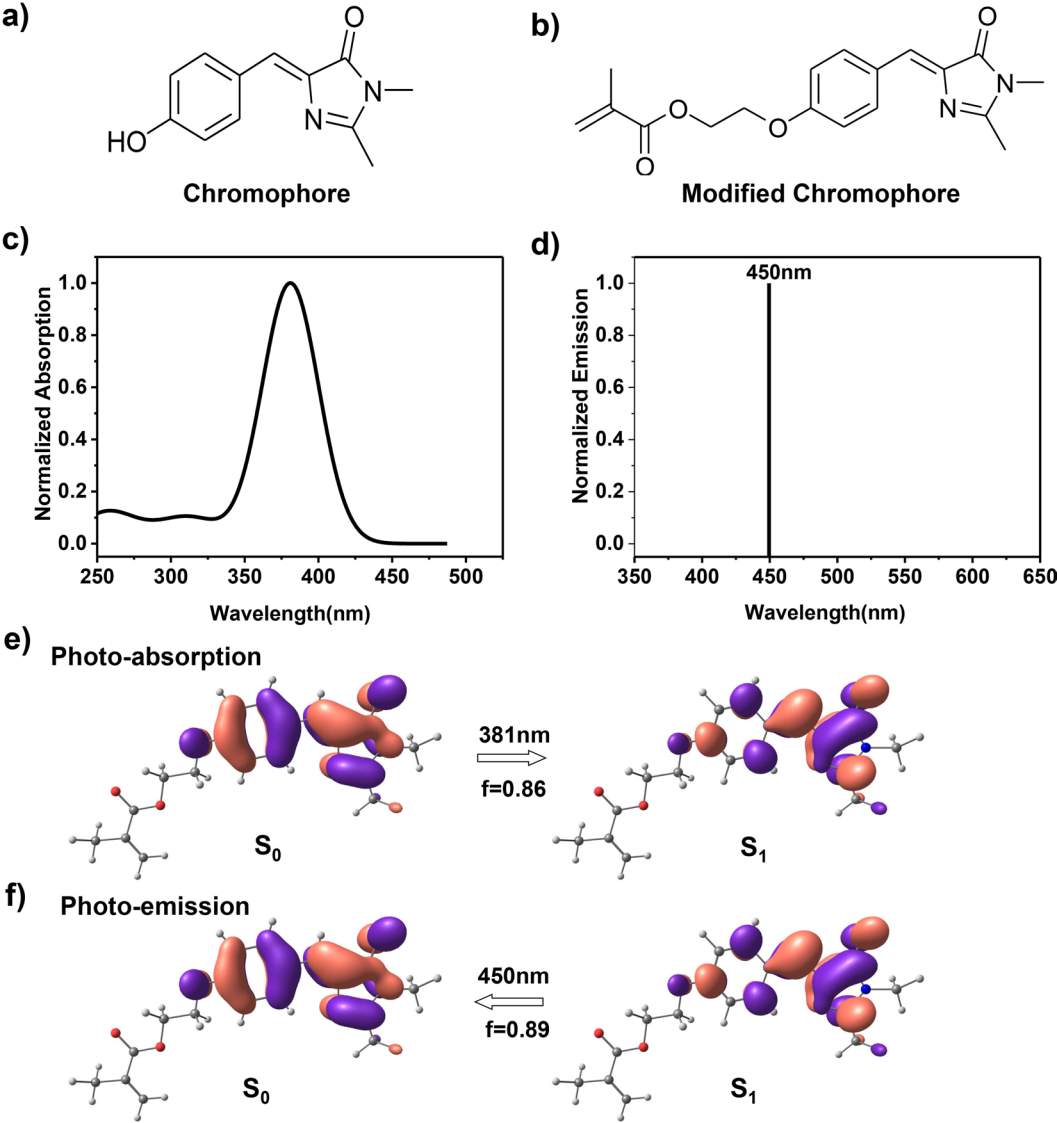
Photo absorption and emission process of the modified GFP chromophore. (**a**) Chemical structures of the GFP chromophore and (**b**) the C=C double bond-modified GFP chromophore. (**c**) The computed absorption spectrum together with the lowest photo-emission peak (**d**) of the modified chromophore. The calculated transitions between frontier orbitals for the photo absorption (**e**) and emission (**f**) of the modified chromophore in aqueous solution. The blue, gray, white and red balls represent N, C, H and O atoms, respectively. f stands for oscillator strength.

The fluorophores responsible for various emission colors from either natural or synthesized fluorescent proteins all share a similar core structure (Fig. 1a). A C=C bond has been introduced into the monomeric chromophore for modification, which is shown in Fig. 1b. Based on the chemical configuration of the modified chromophore, first-principle simulations at the density functional theory (DFT) level were carried out to study the structural information and optical properties. The photo-absorption process was computed after optimizing molecular geometries in the ground state (**S**_**0**_). As depicted in Fig. 1c,d, the main absorption band was found to be a Gaussian peak centered at ~381 nm, while the emission wavelength exhibited a relatively large stokes shift at 450 nm as calculated by the time-dependent density functional theory (TDDFT) method. Our simulations suggested that the modified chromophore in which the natural protein pocket is absent emits blue light instead of green light. Meanwhile, the photo absorption of **S**_**0**_ → **S**_**1**_ and photo emission of **S**_**1**_ → **S**_**0**_ could be ascribe to the frontier orbitals transitions of highest occupied molecular orbital (HOMO) and lowest unoccupied molecular orbital (LUMO) with the oscillator strength of 0.86 and 0.89, respectively (Fig. 1e,f).

**Figure 2:**
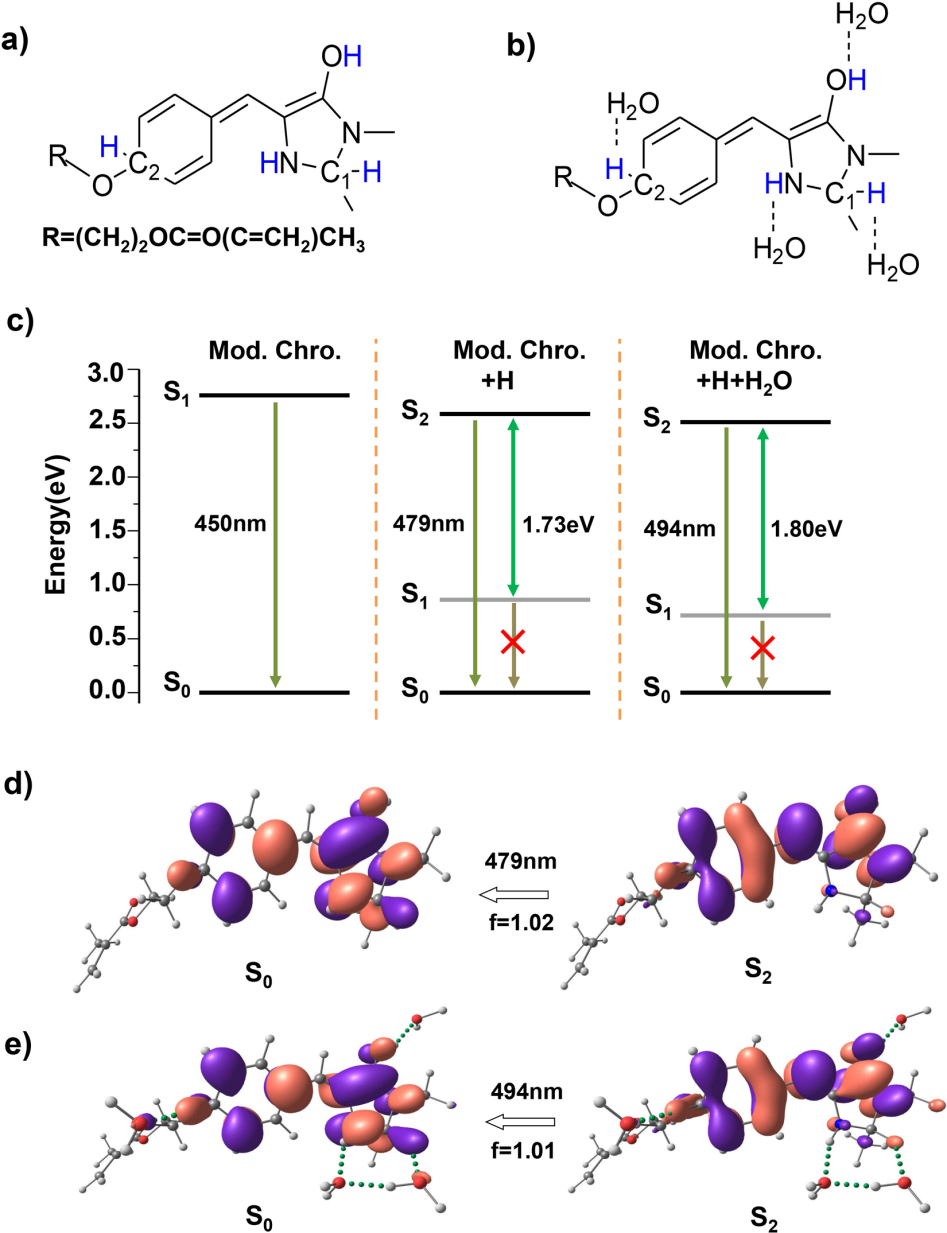
Fluorescent redshift of the modified chromophore with hydrogen bonding network consisting of hydrogen and water. Molecular configurations of the modified chromophore with hydrogen in the (**a**) absence and (**b**) presence of water molecules, added hydrogen atom are highlighted in blue. (**c**) The computed electronic energy levels and photo-emission transitions of the two excited-state structures and the original modified chromophore. The orbital transitions for the calculated photo-emission peak of the chromophore with hydrogen in the (**d**) absence and (**e**) presence of water molecules in aqueous solution. The green dotted bonds denote hydrogen bonding.

In native GFP, only the GFP chromophore is responsible for the fluorescence. Additionally, it is speculated that photo emission could depend strongly on the hydrogen bonding network. Therefore, our theoretical calculation in this work is to investigate the impact of hydrogen bonding consisting of hydrogen (proton) and water on the fluorescence of the GFP-like chromophore. In our simulation, hydrogen was covalently linked to the modified chromophore, while water molecules combine with the hydrogen atoms through hydrogen bonding. Out of various possible structures, the two most stable structures were shown in Fig. 2a,b. These two structures are similar to the protonation states of the GFP chromophore reported in the previous litearture^23^. Analysis of the optimized configurations found that, the presence of hydrogen and water molecules leads to a considerable variation in the overall molecular geometry, especially in the excited-state structures (Fig. S1 in the supplementary information), Furthermore, the electronic structure would also be shifted in response to the change in conjugation, which consequently affects the fluorescent process. Between the two most stable structures, the latter one that bears hydrogen bonds consisting of water molecules and hydrogen is more reasonable, since it is in line with the fact that the practical chromophore would often interacts with water molecules in solution.

The photo-emission processes of these two structures were then simulated at the TDDFT level. Compared to the original chromophore (Fig. 1a), the emission maximum of the structure with hydrogen exhibits a redshift of ~30 nm (479 nm). Water molecules would further cause a redshift of ~15 nm (494 nm) into the green light region. Analysis of molecular orbital transitions and energy levels can reveal the underlying mechanism for the redshift of the fluorescence (Fig. 2c,e). It is noted that the molecular orbitals for the lowest excited states (**S**_**1**_) of the structure with hydrogen are mainly distributed at the side chains regardless of the presence of water (Fig. S2 in the supplementary information). In addition, the gap between the dark state **S**_**1**_ and the bright state **S**_**2**_ is quite large (1.73 eV and 1.80 eV for the chromophore with hydrogen in the presence and absence of water, respectively). This suppresses the internal conversion from **S**_**1**_ to **S**_**2**_ and thus allows for emission of fluorescence breaking the Kasha’s rule^24^. Therefore, as reflected by the zero oscillator strength, the **S**_**1**_ → **S**_**0**_ transition has no contributions to the luminescent process. In contrast, the photoemission generated by the **S**_**2**_ → **S**_**0**_ transition (f = 1.02 and 1.01 for the chromophore with hydrogen in the presence and absence of water, respectively) undergoes a redshift due to the migration of energy levels when the chromophore is combined with hydrogen or further associated with water molecules (Fig. 2c). These photo-emission transitions are consistent with the corresponding absorption transition results (see Fig. S3 in the supplementary information). Compared to the original modified chromophore, it is obvious that the molecular orbitals and the energy levels of the chromophore are altered considerably by the participation of hydrogen and water molecules, which leads to a significant change of emission. In short, this hydrogen bonding network around the GFP-like chromophore changes the electronic structure and charge distribution of the molecule, resulting in the redshift of fluorescence emission.

**Figure 3:**
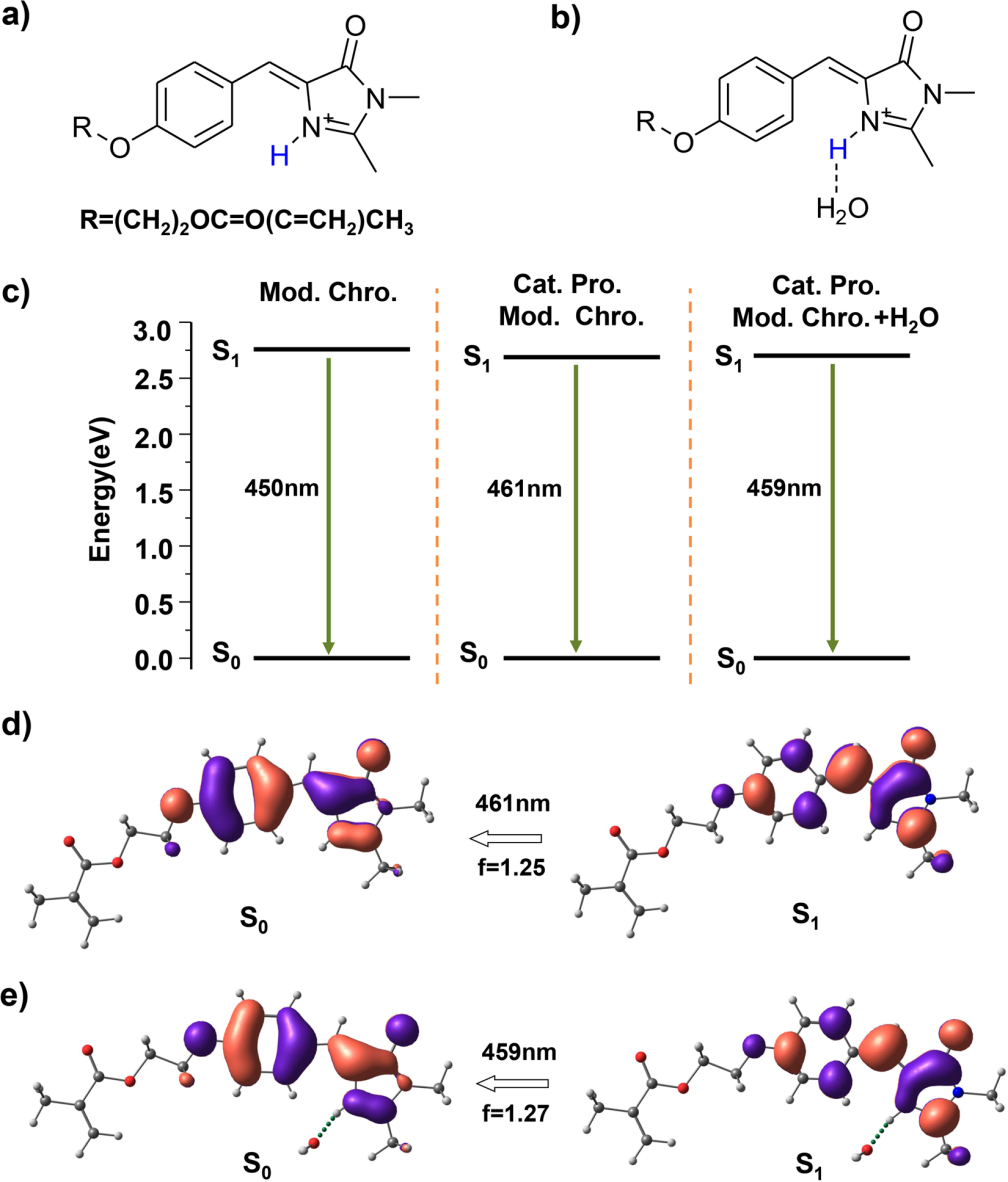
Optical properties of the cationic protonated modified chromophore and that with water molecule. Molecular configurations of the monocationic protonated modified structure in the (**a**) absence and (**b**) presence of water molecule, added protons are highlighted in blue. (**c**) The computed electronic energy levels and photo-emission transitions of these two excited-state structures and the original modified chromophore. The calculated transitions between frontier orbitals for the photo-emission peak of the protonated cation in the (**d**) absence and (**e**) presence of a water molecule.

**Figure 4:**
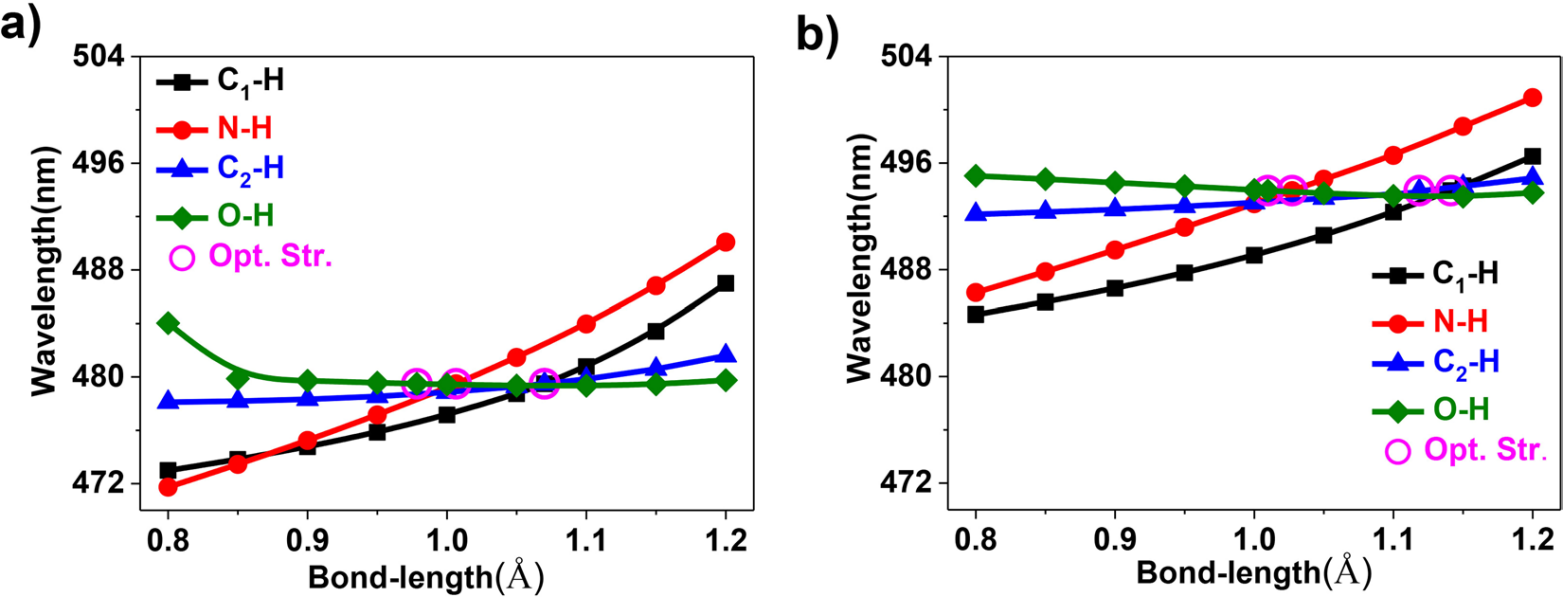
Relationship between the emission wavelengths and relevant bond lengths. The calculated emission maximum as a function of selected bond lengths for the modified chromophore with hydrogen in the (**a**) absence and (**b**) presence of water molecules in aqueous solution.

In addition, the influence of the hydrogen bonding network consisting of proton and water molecules on the photo-emission process of this GFP-like chromophore was also considered. We chose the monocationic structures with protonated imidazole nitrogen in the absence and presence of an associated water molecule to demonstarte it (Fig. 3a,b). The absorption maximum at 402 nm of the cationic protonated structure exhibits a redshift of ~21 nm compared to the original modified chromophore, which is in good agreement with the previous literature (Fig. S4a,b in the supplementary information)^25,26^. However, the emission wavelength at 461 nm only shows a redshift of ~11 nm, suggesting that the cationic structure would still emit blue light (Fig. 4c,d). Further, the addition of a water molecule to the protonated structure hardly leads to a change in the emission wavelength (459 nm, Fig. 4c,e). Electronic **S**_**1**_ → **S**_**0**_ transitions as reflected by wave functions of molecular orbitals in these two structures are similar to the original structure, and the energy levels also barely alter, which gives rise to the small redshift in the emission peak (Fig. 4c–e). These results indicate that the cationic monoprotonated chromophore without significant configuration change couldn’t cause obvious perterbation of the internal electronic structure, thus inducing only a very modest shift of the fluorescent emission peak. Adopting an appropriate configuration tuned by the hydrogen bonding network is important for controlling this system’s luminescencent properties.

Since hydrogen are covalently linked to the chromophore in Fig. 2, we further explored the relationship between the emission wavelengths and selected relevant X-H (X=C, N, O) bond lengths (Fig. 4). It is noted that the emission peaks of the chromophore with hydrogen in the absence and presence of water molecules both show continuously redshift with the increase of C_1_-H, C_2_-H and N-H σ-bond lengths, in which N-H σ-bond length will cause a more pronounced shift. In contrast, increase of the O-H σ-bond length leads to a continuous blue shift of the fluorescence. Moreover, the presence of hydrogen and water molecules may indirectly change the above mentioned σ-bond lengths through hydrogen bonding, which can in turn change the emission wavelength. Accordingly, one can obtain a wide range of desired emission wavelengths for this GFP-like chromophore by tuning the relevant bond lengths. This simple and straightforward approach can be used to modulate the fluorescence wavelength of other GFP species or luminescent molecules.

In summary, to better understand the luminescent mechanism of GFP-like chromophore and modulate the fluorescence process, we investigated the sensitivities of the fluorescence to hydrogen bonding network. We found that hydrogen alter the molecular configuration of the GFP-like chromophore, while water molecules stabilize the hydrogen and regulate the photo emission by hydrogen bonding. The electronic structure of the chromophore has been tuned in response to the change of preferred configurations in the presence of hydrogen bonding network (consisting of hydrogen and water), which gives rise to a redshift of the fluorescent emission. In contrast, the hydrogen bonding with proton and water molecule couldn’t lead to significant fluorescent alteration due to the little change of electronic structure. This provides an efficient knob to control the spectral properties of GFP-related chromophores via hydrogen bonding, thus expanding the applications of GFP-like chromophores and some other relevant fluorescent materials.

In summary, to better understand the luminescent mechanism of GFP-like chromophore and modulate the fluorescence process, we investigated the sensitivities of the fluorescence to hydrogen bonding network. We found that hydrogen alter the molecular configuration of the GFP-like chromophore, while water molecules stabilize the hydrogen and regulate the photo emission by hydrogen bonding. The electronic structure of the chromophore has been tuned in response to the change of preferred configurations in the presence of hydrogen bonding network (consisting of hydrogen and water), which gives rise to a redshift of the fluorescent emission. In contrast, the hydrogen bonding with proton and water molecule couldn’t lead to significant fluorescent alteration due to the little change of electronic structure. This provides an efficient knob to control the spectral properties of GFP-related chromophores via hydrogen bonding, thus expanding the applications of GFP-like chromophores and some other relevant fluorescent materials.

## Methods

### First-principle simulations

All the calculations were performed by using the software package of GAUSSIAN09^27^ at the density functional theory (DFT) level^28^. The hybrid functional B3LYP and the 6–31 + G(d,p) basis were used as it has been confirmed by the previous literature that this level of theory is accurate enough to investigate the optical properties of GFP chromophore^29^. Polarizable continuum model (PCM) was selected to simulate the implicit solution effect^30^. For purposes of geometry optimization and optical process simulation, all the excited states were computed with the time-dependent density functional theory (TDDFT) method^31^.

## Supplementary information

GFP-SciRep-SI-20190510
