# Supplementary material for: Role of Hydrogen Bonding in Green Fluorescent Protein-like Chromophore Emission
Source: Sci Rep. 2019 Aug 12;9:11640. doi: 10.1038/s41598-019-47660-0 (PMC6690883; doi:10.1038/s41598-019-47660-0)
Supplement: Supplementary file 1 — GFP-SciRep-SI-20190510 [file 41598_2019_47660_MOESM1_ESM.docx]

Electronic Supplementary Information for

Role of Hydrogen Bonding in Green Fluorescent Protein-like Chromophore Emission

Li Yang^1,2,†^, Shifeng Nian^2,†^, Guozhen Zhang^2^, Edward Sharman^4^, Hui Miao^2^, Xuepeng Zhang^2,^**^🟊^**, Xiaofeng Chen^3,^**^🟊^**, Yi Luo^2^, Jun Jiang^2,^**^🟊^**

^1^Institutes of Physical Science and Information Technology, Anhui University, Hefei, Anhui 230601, P. R. China.

^2^Hefei National Laboratory for Physical Sciences at the Microscale, iChEM (Collaborative Innovation Centre of Chemistry for Energy Materials), CAS Centre for Excellence in Nanoscience, Department of Chemistry and Materials Science, University of Science and Technology of China, Hefei, Anhui 230026, China.

^3^Department of Environmental Science and Engineering, College of Life and Environmental Science, Shanghai Normal University, Shanghai, 200234, China.

^4^Department of Neurology, University of California, Irvine, California 92697, United States.

^†^These authors contributed equally to this work.

**^🟊^**Corresponding Author

E-mail address: zhangxp@ustc.edu.cn; xiaofengchen@shnu.edu.cn; jiangj1@ustc.edu.cn


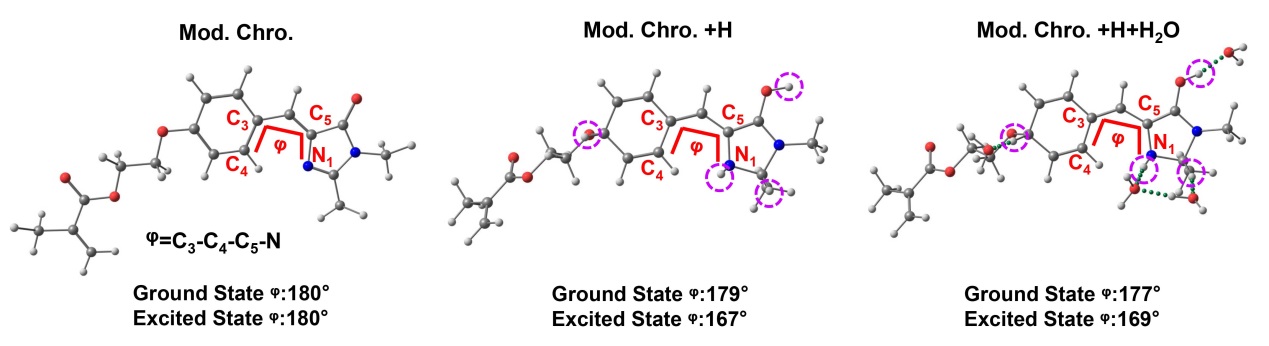


**Figure S1**. Representative optimized configurations showing torsional shifts of the chromophore in three ground/excited-state structures (modified chromophore, modified chromophore with hydrogen in the absence and presence of water molecules). The added hydrogen atoms are highlighted by dotted circles.

In the ground state, there is only a small decrease in dihedral angle between the imidazole ring and benzene ring (a decrease of 1°on going to the structure with hydrogen atoms, and an additional 2° upon hydration), demonstrating only slight planar change. In the excited states, however, the imidazole ring deviates 13° from the planarity of the benzene ring on going to the structure with hydrogen, and 11° for the further water molecules involved.

**
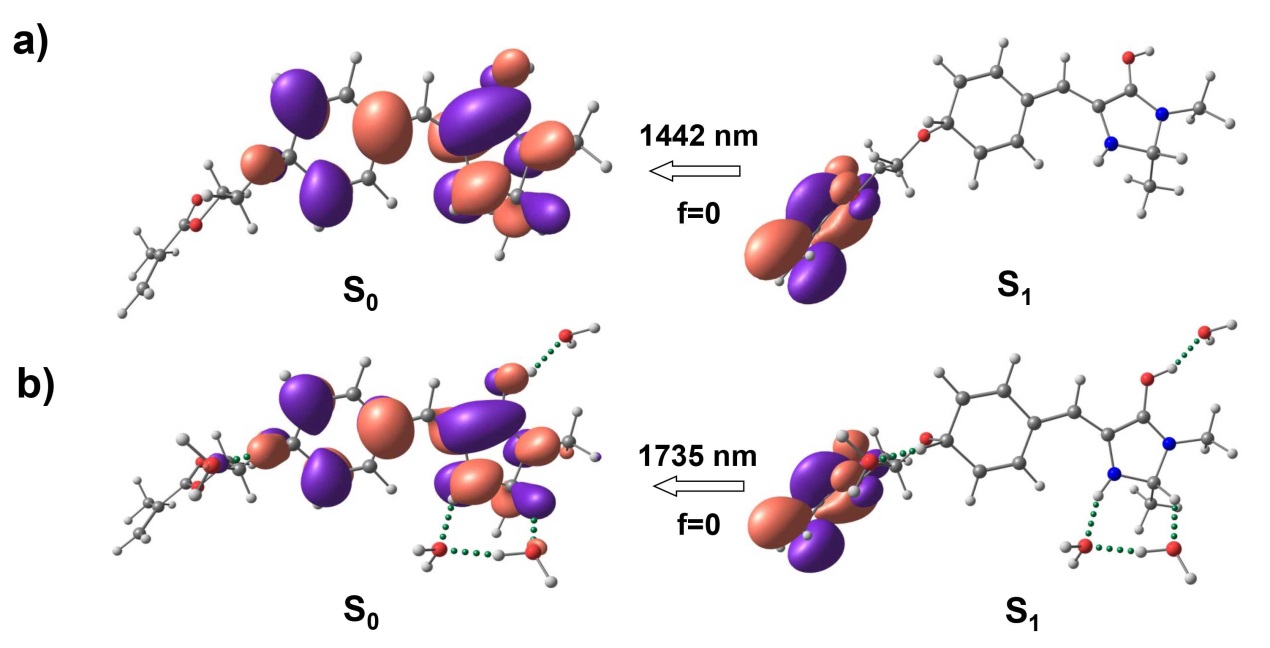
**

**Figure S2**. The orbital transitions for **S_1_→S_0_** of the modified chromophore with hydrogen in the (a) absence and (b) presence of water molecules in aqueous solution. The emission oscillator strengths of these transitions are all zero. The blue, gray, white and red beads stand for N, C, H and O atoms, respectively. The green dotted bonds denote hydrogen bonding.


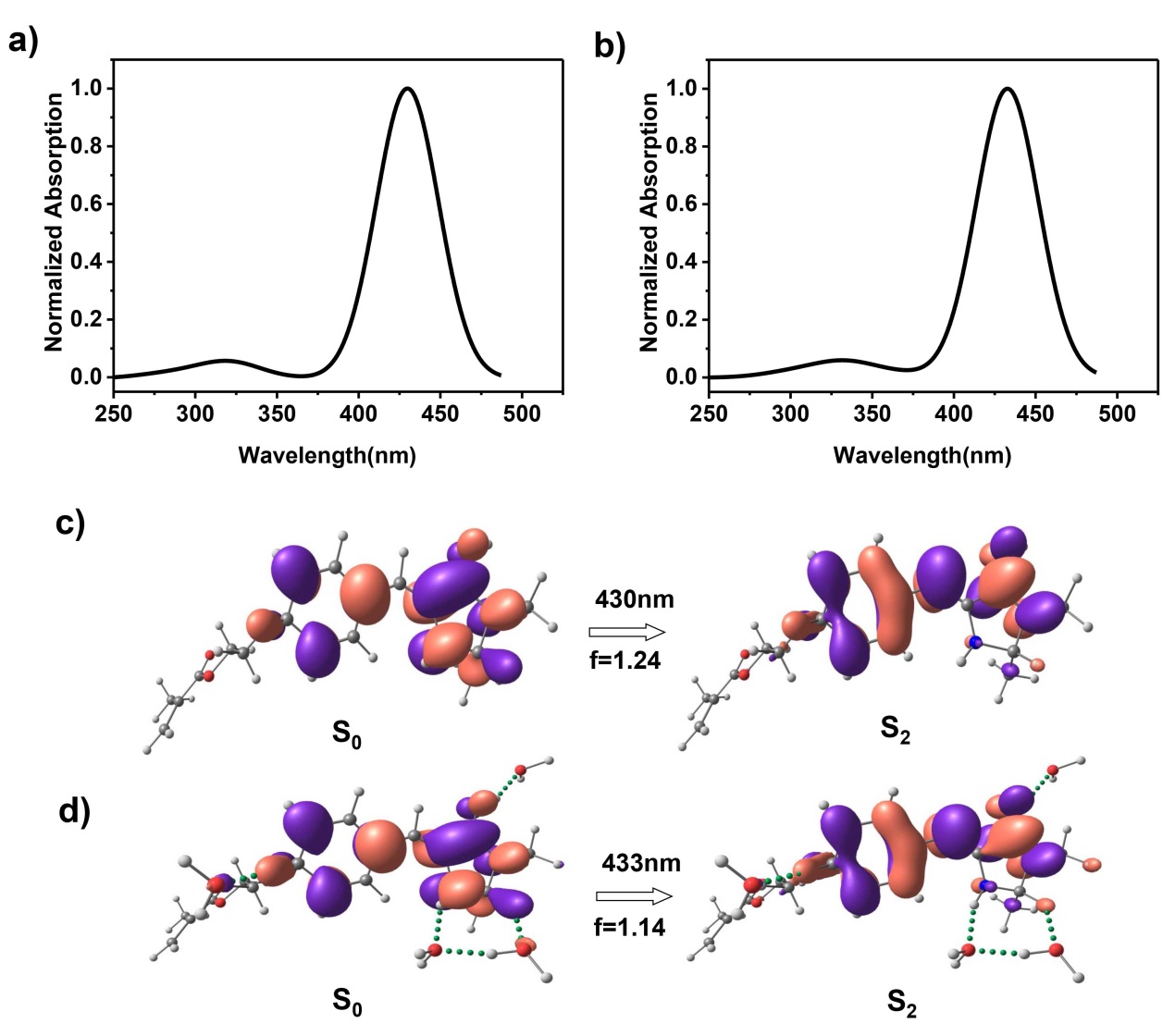


**Figure S3**. Calculated absorption spectrum of the modified chromophore with hydrogen in the (a) absence and (b) presence of water molecules. The orbital transitions for the computed maximum photo-absorption wavelength (first effective absorption peak) of the chromophore with hydrogen in the (c) absence and (d) presence of water molecules in aqueous solution.


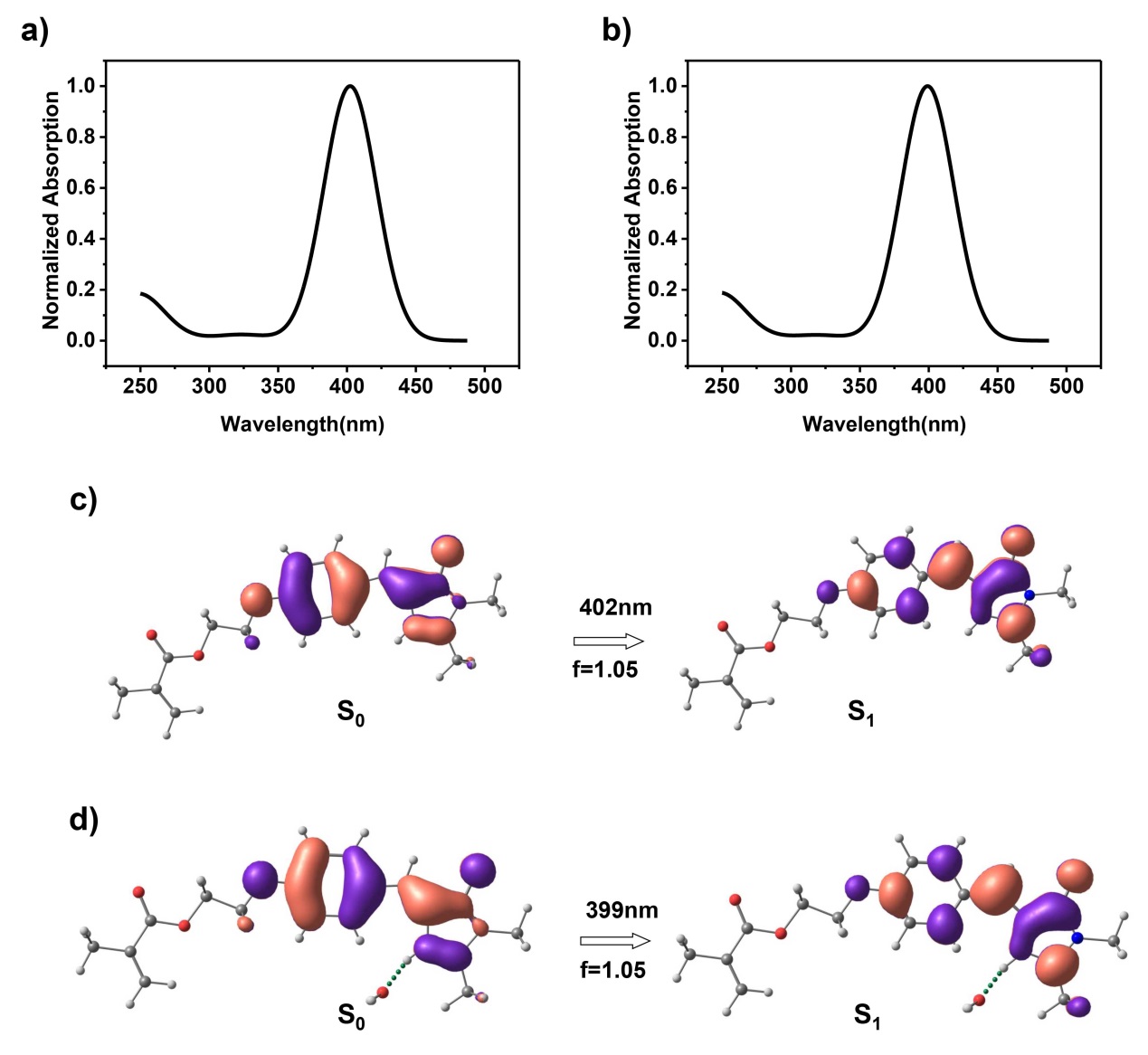


**Figure S4**. The computed absorption spectrum of the cationic protonated structures in the (a) absence and (b) presence of water molecule. The calculated transitions between frontier orbitals for the photo absorption of the protonated cation in the (c) absence and (d) presence of water molecule.
